# The utility of telemedicine in managing patients after COVID-19

**DOI:** 10.1038/s41598-022-25348-2

**Published:** 2022-12-10

**Authors:** Krystian T. Bartczak, Joanna Milkowska-Dymanowska, Wojciech J. Piotrowski, Adam J. Bialas

**Affiliations:** 1 Department of Pneumology, Medical University of Lodz, Kopcinskiego 22, 90-153 Lodz, Poland; 2 Department of Pulmonary Rehabilitation, Center for Lung Diseases and Rehabilitation, Blessed Rafal Chylinski Memorial Hospital for Lung Diseases, Lodz, Poland

**Keywords:** Diseases, Health care, Medical research

## Abstract

Despite growing knowledge about transmission and relatively wide access to prophylaxis, the world is still facing a severe acute respiratory syndrome coronavirus 2 (SARS CoV 2) global pandemic. Under these circumstances telemedicine emerges as a powerful tool for safe at-home surveillance after a hospital discharge; the data on when to safely release a patient after acute COVID-19 is scarce. Reckoning an urgent need for improving outpatient management and possibly fatal complications of the post-COVID period, we performed the pilot telemonitoring program described below. The study aimed to assess the usefulness of parameters and surveys remotely obtained from COVID-19 convalescents in their individual prognosis prediction. Patients were involved in the study between December 2020 and May 2021. Recruitment was performed either during the hospital discharge (those hospitalized in a Barlicki Memorial Hospital in Lodz) or the first outpatient visit up to 6 weeks after discharge from another center. Every participant received equipment for daily saturation and heart rate measurement coupled with a tablet for remote data transmission. The measurements were made after at least fifteen minutes of rest in a sitting position without oxygen supplementation. Along with the measurements, the cough and dyspnea daily surveys (1–5 points) and Fatigue Assessment Scale weekly surveys were filled. We expected a saturation decrease during thromboembolic events, infectious complications, etc. A total of 30 patients were monitored for a minimum period of 45 days, at least 2 weeks after spontaneous saturation normalization. The mean age was 55 (mean 55.23; SD ± 10.64 years). The group was divided according to clinical improvement defined as the ≥ 10% functional vital capacity (FVC) raise or ≥ 15% lung transfer for carbon monoxide (T_L,CO_) rise. Our findings suggest that at-rest home saturation measurements below 94% (*p* = 0.03) correspond with the lack of clinical improvement in post-COVID observation (*p* = 0.03). The non-improvement group presented with a lower mean—94 (93–96)% versus 96 (95–97)%, *p* = 0.01 and minimum saturation—89 (86–92)% versus 92 (90–94)%, *p* = 0.04. They also presented higher variations in saturation measurements; saturation amplitude was 9 (7–11)% versus 7 (4–8)%, *p* = 0.03; up to day 22 most of the saturation differences reached statistical significance. Last but not least, we discovered that participants missing 2 or more measurements during the observation were more often ranked into the clinical improvement group (*p* = 0.01). Heart rate day-to-day measurements did not differ between both groups; gathered data about dyspnea and cough intensity did not reach statistical significance either. A better understanding of the disease’s natural history will ultimately lead us to a better understanding of long COVID symptoms and corresponding threats. In this paper, we have found home oxygen saturation telemonitoring to be useful in the prediction of the trajectory of the disease course. Our findings suggest that detection of at-rest home saturation measurement equal to or below 94% corresponds with the lack of clinical improvement at the time of observation and this group of patients presented higher variability of day-to-day oxygen saturation measurements. The determination of which patient should be involved in telemedicine programs after discharge requests further research.

## Introduction

Despite growing knowledge about transmission and relatively wide access to prophylaxis, the world is still facing a severe acute respiratory syndrome coronavirus 2 (SARS CoV 2) global pandemic. With every coronavirus disease 2019 (COVID-19) outbreak hospitals are filling with worsening patients and it forces the already stable and treated individuals out of the hospital. Part of them still suffer from hypoxemia and require further oxygen treatment at home. The need for optimization of resources has been noticed both by medics and politicians, while telemedicine can be a powerful tool for safe at-home supervision and treatment^1^.

The knowledge about symptoms and natural history post-COVID is continuously evolving^2^. Available research usually assessed patients’ condition after a specific time from hospital discharge; little is known about their respiratory insufficiency between discharge and the first outpatient assessment^3^. To our knowledge, there is no evidence-based data on when to safely release a patient from the hospital^4^.

Telemedicine is defined as “the use of electronic information and communications technologies to provide and support health care when distance separates the participants”^5^. Telehealth measures are proven to be effective in controlling the epidemic, as well as improving patients’ safety and treatment, especially for those living in remote regions^6^. Reckoning an urgent need for improving outpatient management and possibly fatal complications after hospital discharge, we started the pilot telemonitoring program described below. The study aimed to assess the usefulness of simple life parameters and surveys remotely obtained from patients after acute COVID-19 in predicting future outcomes. Additionally, we wanted to evaluate the time required from hospital release to percutaneous saturation normalization and to identify health complications of the recovery period.

## Methods

This prospective observational study was performed in the Outpatient Pneumology Clinic of The Medical University of Lodz. It has been approved by the Bioethical Committee of the Medical University of Lodz (No. RNN/58/21/KE). All research was performed in accordance with relevant guidelines and regulations, including the Declaration of Helsinki. Participants recruited for the study between December 2020 and May 2021 were patients after acute interstitial COVID-19 pneumonia either leaving the pneumology unit of Barlicki Memorial Teaching Hospital of the Medical University of Lodz or patients visiting the outpatient pneumology clinic of Teaching Hospital No. 1 up to six weeks after discharge from other centers. Participants were equipped with telemedicine equipment consisting of pulse oximeters (MIR Spirotel®) connected with Samsung Galaxy Tab 3 tablets; the set was equipped with an internet SIM card and associated with a web application Atencare (Mediguard®). Everyone underwent the 15-min technical training and informed consent was obtained from all subjects. We included adult convalescents that were previously hospitalized from COVID-19 pneumonia and agreed to participate in the study. The exclusion criteria featured patients unable to operate the equipment and provide data for analysis, those requiring mechanical ventilation during the acute pneumonia phase, and those with preexisting respiratory failure or interstitial lung diseases.

Every day the participants were supposed to evaluate their saturation and heart rate with a minimum of 2-min percutaneous measurement; always performed at rest with no oxygen supplementation for at least 15 min. The intensity of cough and dyspnea was estimated in simple Likert 1 to 5 points scale daily surveys^7,8^. The results were analyzed daily and participants were able to reach the physician-in-charge at any time with a phone call to address new/worsening symptoms and assess the urgency of medical intervention. This possibility was intended to detect possible medical complications during the study course without unnecessary delay.

At the same time, the clinical examination of participants was performed twice; first between two to six weeks after hospital discharge and second after two consecutive months. Apart from physical examination, pulmonary function tests (PFTs) such as spirometry, lung transfer for carbon dioxide (T_L,CO_), and 6-min walk test (6MWT) have been performed. The group was further divided for analysis according to clinical improvement defined as the ≥ 10% functional vital capacity (FVC) rise or ≥ 15% lung transfer for carbon monoxide (T_L,CO_) rise between appointments. The improvement criteria for FVC and TL,CO are reflected from several studies which prove that such an alteration in these parameters is associated with different outcomes in interstitial lung diseases (ILDs) like e.g. idiopathic pulmonary fibrosis^9–11^ and scleroderma-associated ILD^12,13^.

Statistical analysis was performed using R software for macOS. Continuous data were presented as the mean with SD or median with interquartile range (IQR), depending on the distribution of data. Variables were compared using the unpaired Student’s t-test, Welch t-test, or the Wilcoxon rank-sum test with continuity correction, depending on data normality and homogeneity of variance. Categorical data were analyzed using the Chi-square test or Fisher’s Exact Test, based on assumptions of the tests. Missing data was not imputed in the analysis.

## Results

Thirty patients with a mean age of 55 (mean 55.23; SD ± 10.64 years) were involved in the study. Participants’ characteristics are presented in Table 1. Our findings suggest that at-rest home saturation measurements below 94% (*p* = 0.03) correspond with the lack of clinical improvement in post-COVID observation (*p* = 0.03). The non-improvement group presented with a lower mean—94 (93–96) % versus 96 (95–97)%, *p* = 0.01 and minimum saturation—89 (86–92)% versus 92 (90–94)%, *p* = 0.04. They also presented higher variations in saturation measurements; saturation amplitude was 9 (7–11)% versus 7 (4–8)%, *p* = 0.03. Until the 22nd monitoring day, most of the saturation differences reached statistical significance. The comparison of both study groups along with statistical values is specified in Table 2. Last but not least, we discovered that participants missing 2 or more measurements were more often ranked into the clinical improvement group (*p* = 0.01). Heart rate day-to-day measurements, as well as survey data about dyspnea and cough intensity, did not differ between both groups. Alterations among previous COVID-19 pneumonia treatment and comorbidities did not reach statistical significance either.

**Table 1:** Characteristics of study participants.

Parameter	Value
Age, years, mean (SD)	55.23 (10.64)
Male, n (%)	23/30 (76.66)
BMI, kg/m^2^, mean (SD)	28.85 (5.12)
Packyears, median [IQR]	15 [8–30]
Hospitalization days, median [IQR]	12 [7–26.25]
CT initial lung involvement 0–4 scale, points [IQR]	3 [2–4]
Invasive ventilation, n	0
Non-invasive ventilation, n	4
HFNOT, n	9
Mean initial saturation, %, mean (SD)	95.53 (1.79)
Mean initial heart rate, bpm, mean (SD)	78.48 (8.62)
Mean initial dyspnea, 1–5 visual scale, points, mean (SD)	1.70 (0.70)
Mean initial cough, 1–5 visual scale, points (SD)	1.43 (0.62)
FEV1, L, mean (SD)	2.75 (1.01)
FEV1, %, mean (SD)	78.15 (20.36)
FVC, L, mean (SD)	3.36 (1.32)
FVC, %, mean (SD)	75 (21.27)
TLC, L, median, [IQR]	5.04 [4.24–6.75]
TLC, %, mean, (SD)	79.96 (24.93)
T_L,CO_, mL/min/mm Hg, mean, (SD)	6.27 (2.72)
T_L,CO_, %, mean, (SD)	73.37 (27.98)

*BMI* body mass index, *CT* computed tomography, *FEV1* forced expiratory volume in 1 s, *FVC* forced vital capacity, *HFNOT* high flow nasal oxygen cannula, *TLC* total lung capacity, *T*_*L,CO*_ transfer factor of the lung for carbon monoxide.

**Table 2:** Comparison of the improvement and non-improvement participant groups.

Parameter	Improvement (n = 20)	Non-improvement (n = 10)	*p* value
Saturation amplitude, %, median [IQR]	7 [4–8]	9 [7–11]	0.03
Mean saturation, %, mean (SD)	96 (1)	94 (2)	0.01
Minimum saturation, %, mean (SD)	92 (4)	89 (3)	0.04
Hospitalization time, days, median [IQR]	12 [7–14]	30 [15–48]	0.008
CT initial score, points, median [IQR]	2/4 [1–3]	3/4 [3–4]	0.008
CT control score, points, median [IQR]	¼ [1–2]	4/4 [3–4]	0.00002
FEV1 I, %, mean (SD)	87.28 (13.67)	59.89 (19.69)	0.0003
FEV1 II, %, mean (SD)	90.22 (9.39)	69.1 (16.39)	0.0002
FVC I, %, mean (SD)	84.44 (14.01)	56.11 (21.23)	0.0003
FVC II, %, mean (SD)	88.11 (9.87)	62.7 (15.32)	0.00001
FEV1/FVC I, mean (SD)	82.1 (7.51)	84.78 (6.69)	0.38
FEV1/FVC II, mean (SD)	80.7 (4.24.)	86.26 (6.41)	0.01
TLC I, %, mean (SD)	89.56 (16.03)	60.78 (29.2)	0.003
TLC II, %, mean (SD)	93 (13.38)	75.6 (18.3)	0.008
T_L,CO_ I, %, mean (SD)	80.11 (21.93)	57.38 (35.42)	0.05
T_L,CO_ II, %, mean (SD)	90 (19.31)	68.9 (27.93)	0.03

Pulmonary function tests are presented during the first (I) and second (II) assessments.

*CT* computed tomography, *FEV1* forced expiratory volume in 1 s, *FVC* forced vital capacity, *TLC* total lung capacity, *T*_*L,CO*_ transfer factor of the lung for carbon monoxide.

## Discussion

Telesolutions have been increasingly developing during past epidemics, e.g., for contact investigations and disease control^6^. In the time of the Ebola outbreak between the years 2014 and 2016, a mobile application helped to trace and monitor confirmed cases^14^, increasing the time to case registration, completeness, and security of the data. When SARS-CoV 1 was dealt with in Taiwan, online communication via a webcam increased the availability of medical consultations and reduced their costs^15^. Swiss teleservice notifications associated with fever were proven to reflect influenza activity^16^.

Lately, the pandemic situation forced outpatient care out of the doctor’s office. Italian authors, like Omboni^17^, complained of insufficient telemedicine implementation during the first striking COVID-19 wave and suggested it is a must in a modern healthcare system, especially in terms of chronically-ill patients during a lockdown. Nevertheless, more attention in the literature has been put on telehealth concerning home-isolated COVID-19 patients and controlling their disease^18^, especially after the unpredictable character of SARS-CoV 2-associated pneumonia presented itself throughout the world. The China health center^19^ has set up a network for COVID-19 alert and response with 126 network hospitals involved. Between January 28 and February 17 in 2020 63 teleconsulted patients had severe pneumonia alongside 591 were moderate cases. At that time, there were mobile devices used for collecting, evaluating, and reporting patient vital signs to the caring team in isolation wards. The authors outline that the bedside system allowed to limit the exposure to patients’ contagious secretions and the communication system was utilized to build and remotely train multidisciplinary health teams for a more comprehensive treatment. An utterly different telesurveillance solution for home-treated patients based on questionnaires in a mobile application has been developed by a French group^20^, with more than 65,000 users in Paris, and it consisted of Medical Responders and physicians reacting to changing subjective health status of ill individuals.

The effects of telemedicine implementation and its impact are well known in cardiology, with trials such as the TIM-HF2^21^ which demonstrated that remote telemonitoring reduced days lost due to unplanned cardiovascular hospitalizations, as well as documented a decrease in all-cause mortality among patients managed in the study. In chronic obstructive pulmonary disease, our research group has previously shown that a decrease in saturation exceeding 4% can predict an exacerbation in the forthcoming 7 days^22^. With enough time and training, home spirometry starts to correlate with hospital spirometry among the idiopathic pulmonary fibrosis group^23^. Among the less obvious effects of telemedicine-enhanced home care, the worth-to-consider effects are both improved psychological well-being and individually tailored treatment decisions.

Much is known about the average recovery after COVID-19 pneumonia and persistent symptoms; however, the variety of research does not answer the question of which patients should be supervised with special attention. To our knowledge, no other group monitored patients at home for a longer period than we managed to do. This study aimed to stratify the usefulness of day-to-day saturation and heart rate measurements, as well as the subjective extent of dyspnea and cough, in post-COVID care among previously hospitalized survivors. Our findings suggest that a patient who provides at-rest saturation measurements lower than 94% will not significantly improve in pulmonary function tests—FVC and DLCO—after 2 to 3 months post-discharge. This equals continuous exercise intolerance.

Most of the existing research associated with COVID-19 and telehealth considers the acute infection stage, sometimes with a short sequence afterward. Motta et al. monitored saturation, heart rate, body temperature and peak expiratory flow of 12 patients during 30 consecutive days of acute home-treated individuals with mild to asymptomatic SARS CoV 2 infestations to evaluate a quick response system during the worsening symptoms^24^. The findings have shown a significant decrease in SpO2 and an increase in heart rate during the illness, while PEF values dropped below 80% of the normal range among 4 of the participants. The authors outlined that there was not enough data to guide the use of home pulse oximetry or validate it in disease progression.

O’Carroll et collaborates used remote oxygen saturation monitoring in COVID-19 cases to facilitate the discharge of non-oxygen-dependent patients and have their safe follow-up^25^. During the median time of 12 days of measurements, the telemonitoring allowed to detect 3/18 patients with desaturations because of worsening COVID-19 infiltrates and 1/18 worsening from hospital-acquired pneumonia developing after the hospital discharge. Telemedicine served its role—it allowed managing patients’ conditions in a more controlled manner; no one from this group (4/18) required non-invasive or invasive ventilation during readmission. Of note, the alerts that lead to medical attention were programmed to be generated after every measurement lower than 94% SpO_2_, consistent with our findings. The frequency of measurements was higher (mean 3.9 vs 5.7 per day) in the readmission group^25^; our own observations showed a higher cooperation rate among more seriously ill (non-improvers). Similar research from Grutters et al. proved the 5-day (± 3.8) shortening of hospital stay among the 33 participants group via the use of telehealth. It also allowed a safe follow-up with 3 readmissions and 1 pulmonary embolism diagnosis, along with the cost-effectiveness of a whole system. Most patients in these studies rate telemonitoring to be friendly and useful.

Research by Martínez-García et al. included two groups of patients in the surveillance^26^: 224 outpatients traced from the beginning of the disease and 89 inpatients after discharge. Every patient provided oxygen saturation and temperature 3 times a day. Proactively, the patient was reached at least once a day. Until the termination of the study after 30 days, 38 (16.90%) outpatients were referred to the Emergency Department, 18 were hospitalized (8.03%), and 2 were deceased. One patient from the inpatient group was re-hospitalized and one left the study. Importantly, neither deaths nor vital emergencies happened at home. The average time of monitoring was 11.64 (± 3.58) days, and 224 (73.68%) patients were discharged during the 30 days of the study.

Patients are reluctant to participate in telehealth research for various reasons explored e.g. by Sanders et al.^27^. Most anxiety comes from technical requirements—which are often misunderstood and exaggerated. There is a group of patients that consider telemedical surveillance with a high degree of dependency and ill health, which is unbearable to them. At the same time, others are glad to have their current healthcare providers and they are hesitant about the care methods they are unfamiliar with. Great telemonitoring adherence data comes from the paper by Lang et al.^28^. Among the analyzed group, some participants withdrew from the study during its course—referred to as the drop-outs. 41 patients gave reasons for dropping out after a period of sending data. They can be further categorized into groups: no perceived benefits for health; no need for telemonitoring; investing too much time in participation; insufficient user-friendliness; feeling a loss of privacy. The most mentioned reasons for dropping out were no perceived benefit (19/41; 46.3%) and the lack of telemonitoring needs (18/41; 43.9%). Cook^29^ also outline that the majority of users resigning from telehealth did not find the equipment useful once they had tried it, while Foster’s^30^ telehealth engagement study reported that as much as 40.1% (n = 2852) of decliners did not feel a need for additional health support, 27.2% (n = 1932) stated being too busy to use it and 15.3% (n = 1092) of decliners were not interested in the research. This data is greatly consistent with our findings, where non-adherence and omitting the daily measurements are correlated with functional improvement after COVID-19. We did not investigate participants’ motivation though, so we can only speculate that they did not feel the necessity to stay under strict surveillance.

Compared to the research cited above, our prospective study is unique because we prolonged the monitoring until a minimum of 2 weeks of SpO_2_ ≥ 95% with a mean observation time of 67 (range 45–114) days. The program allowed us to notice serious events in patients’ individual post-COVID history. It becomes crucial when you realize a striking study by Chopra et al.^3^ who depicted that from 1250 COVID-19 survivors in the US State of Michigan 84 patients (6.7% of hospital survivors and 10.4% of ICU-treated hospital survivors) died in the following 60 days, bringing the overall mortality rate for the cohort to 29.2% hospitalized and 63.5% of treated in ICUs. Data from Bellan et al. further confirm these results with 5% post-discharge 30-day mortality^31^. Furthermore, 189 convalescents (15.1%) became re-hospitalized in the same period. In our group, one of the patients returned to a hospital during the study because of *Clostridioides difficile* diarrhea as a post-hospitalization and post-antibiotic consequence. At the time of observation, the additional diagnoses were: one outpatient post-COVID pulmonary embolism; one hereditary thrombophilia (in another person); two asthma diagnoses; urinary bladder ulcers of possible viral etiology in one patient; myocarditis (two suspected and one of them confirmed in the MRI). There was also one underlying interstitial lung disease suspected but the final diagnosis of severe emphysema with overlapping post-COVID radiological changes has been determined. None of the participants died or had rapidly worsening respiratory parameters. Worthy of note, those hospitalized in pulmonary rehabilitation units reported notable subjective improvement. The efficacy of post-COVID pulmonary rehabilitation is undoubtedly beneficial in research papers^32^. Such a statement cannot be assigned to pharmacological interventions so far.

On the other hand, the heart rate measurements did not prove to be useful in our real-life telemonitoring study, probably because compensatory tachycardia was deeply modified by the use of medications like β-blockers and ivabradine. We were also disappointed with dyspnea and cough self-assessment scales that did not correspond with pulmonary improvement. Interestingly, it appears from existing studies that there is no significant difference in PFTs when comparing patients with persistent COVID-19-related symptoms and asymptomatic ones^33^.

The study could not consider confounding factors. The main limitation of our research is group heterogeneity; as explained in the methods section almost every patient hospitalized for COVID-19 pneumonia was allowed to join it. The severity of interstitial pneumonia among participants was not equal and men were the predominant sex. The starting point slightly differed between patients hospitalized in our unit and those from other centers, forming a possible bias. 6-min walk tests were performed with different supervisors and it probably had an impact on patients’ engagement in the test itself. The unique benefits come from a longer observation time and addressing the pulmonary function tests to pulse oximetry results; as far as we are concerned no other researchers found such a correlation. Every patient had technical training with access to technical and medical consultation whenever problems occurred; just to eliminate loss of data or potentially hazardous events.

## Conclusions

A better understanding of the disease’s natural history will ultimately lead us to a better understanding of long COVID symptoms and corresponding threats. In this paper, we have found home oxygen saturation telemonitoring to be useful in disease course prediction. Our findings suggest that detection of at-rest home saturation measurement equal to or below 94% corresponds with the lack of clinical improvement and that this group of convalescents presents a higher variability of day-to-day oxygen saturation measurements. Convalescents with correct measurements could return to their general practitioners more quickly as they seem to spontaneously recover with time. The determination of which post-COVID patient should be involved in telemedicine programs requires further research.

## Data Availability

The datasets analyzed during the current study are available from the corresponding author upon reasonable request.
